# Increased volume of cerebral oedema is associated with risk of acute seizure activity and adverse neurological outcomes in encephalitis – regional and volumetric analysis in a multi-centre cohort

**DOI:** 10.1186/s12883-022-02926-5

**Published:** 2022-11-07

**Authors:** A. M. Alam, J. P. K. Chen, G. K. Wood, B. Facer, M. Bhojak, K. Das, S. Defres, A. Marson, J. Granerod, D. Brown, R. H. Thomas, S. S. Keller, T. Solomon, B. D. Michael

**Affiliations:** 1 Department of Clinical Infection Microbiology and Immunology, Institute of Infection, Veterinary, and Ecological Science, University of Liverpool, Liverpool, UK; 2 The NIHR Health Protection Research Unit for Emerging and Zoonotic Infection, Liverpool, UK; 3 Barts Health NHS Trust, London, UK; 4 Department of Pharmacology and Therapeutics, Institute of Systems, Molecular and Integrative Biology, University of Liverpool, Liverpool, UK; 5 Department of Neuroradiology, The Walton Centre NHS Foundation Trust, Liverpool, UK; 6 Tropical and Infectious Diseases Unit, Liverpool University Hospitals NHS Trust, Liverpool, UK; 7 Department of Neurology, The Walton Centre NHS Foundation Trust, Liverpool, UK; 8 Independent Scientific Consultant, formerly of Public Health England, London, UK; 9 UK Heath Security Agency, 61 Colindale Avenue, London, UK; 10 Translational and Clinical Research Institute, Faculty of Medical Sciences, Newcastle University, Newcastle upon Tyne, UK

**Keywords:** Encephalitis, Acute encephalitis syndrome, Seizures, Magnetic resonance imaging, Neuroimaging

## Abstract

**Background:**

Seizures can occur unpredictably in patients with acute encephalitis syndrome (AES), and many suffer from poor long-term neurological sequelae. Establishing factors associated with acute seizures risk and poor outcomes could support clinical care. We aimed to conduct regional and volumetric analysis of cerebral oedema on magnetic resonance imaging (MRI) in patients with AES. We assessed the relationship of brain oedema with acute seizure activity and long-term neurological outcome.

**Methods:**

In a multi-centre cohort study, adults and children presenting with an AES were recruited in the UK. The clinical and brain MRI data were retrospectively reviewed. The outcomes variables were inpatient acute seizure activity and neurological disability at six-months post-discharge. A poor outcome was defined as a Glasgow outcome score (GOS) of 1–3. We quantified regional brain oedema on MRI through stereological examination of T2-weighted images using established methodology by independent and blinded assessors. Clinical and neuroimaging variables were analysed by multivariate logistic regression to assess for correlation with acute seizure activity and outcome.

**Results:**

The study cohort comprised 69 patients (mean age 31.8 years; 53.6% female), of whom 41 (59.4%) had acute seizures as inpatients. A higher Glasgow coma scale (GCS) score on admission was a negative predictor of seizures (OR 0.61 [0.46–0.83], *p* = 0.001). Even correcting for GCS on admission, the presence of cortical oedema was a significant risk factor for acute seizure activity (OR 5.48 [1.62–18.51], *p* = 0.006) and greater volume of cerebral oedema in these cortical structures increased the risk of acute seizures (OR 1.90 [1.12–3.21], *p* = 0.017). At six-month post-discharge, 21 (30.4%) had a poor neurological outcome. Herpes simplex virus encephalitis was associated with higher risk of poor outcomes in univariate analysis (OR 3.92 [1.08–14.20], *p* = 0.038). When controlling for aetiology, increased volume of cerebral oedema was an independent risk factor for adverse neurological outcome at 6 months (OR 1.73 [1.06–2.83], *p* = 0.027).

**Conclusions:**

Both the presence and degree of cerebral oedema on MRIs of patients with AES may help identify patients at risk of acute seizure activity and subsequent long-term morbidity.

**Supplementary Information:**

The online version contains supplementary material available at 10.1186/s12883-022-02926-5.

## Background

Encephalitis is inflammation of the brain parenchyma, most commonly caused either by an infectious agent or an autoimmune process [1, 2]. Patients present clinically with an acute encephalitis syndrome (AES) as defined by the World Health Organisation (WHO) [3, 4], which is a combination of fever or coryzal illness and alterations in behaviour, personality, consciousness, or seizure activity. Adverse outcomes are common in AES, with more than half of patients failing to make a full recovery 2 years following their diagnosis [5, 6]. Seizures occur unpredictably in up to two thirds of patients and are associated with increased morbidity and mortality [7–10].

An ability to establish the factors associated with acute seizures risk and poor neurological outcomes in patients with AES could support clinical care. Identifying those at high risk of seizures can guide management decisions, such as determining who will require neurological intensive care support and consideration of electroencephalographic (EEG) monitoring. Moreover, this may support the stratification of AES patients for future clinical trials of anti-seizure medications [11]. Patients with AES are seldom discharged with plans for rehabilitation, despite the majority suffering from long-term sequelae [6, 12]. Early identification of patients at risk of poor outcomes can facilitate early support with their recovery and appropriate referral for longer-term care [12].

Neuroimaging, especially magnetic resonance imaging (MRI), has a critical role in the evaluation of AES and may help in prognostication [13]. Whilst the MRI may be normal or near normal in some aetiologies of encephalitis due to specific autoantibodies (such as those directed at N-methyl-D-receptors) despite prominent seizure activity; overall the presence of observable changes on neuroimaging in patients with AES has been associated with both occurrence of seizures and adverse outcomes. However, it is currently unclear which brain regions and by what degree oedema is most predictive of acute seizure activity [7, 14, 15]. Feasibility studies have shown that it is possible to quantify regional brain oedema in encephalitis by using a stereological point-counting technique on MRI [16].

As an MRI can be obtained early in clinical management (often before the aetiological diagnosis is established and as in many cases no aetiology is ultimately determined), our study evaluated the regions and volume of vasogenic oedema visualised on the brain MRI scan in a well-defined cohort of patients presenting with AES in a multi-centre study. We aimed to assess the role of cerebral oedema on MRI in predicting acute seizure activity and long-term neurological outcome in patients with AES.

## Methods

### Patient recruitment and inclusion/exclusion criteria

We conducted a retrospective study of patients recruited through the Aetiological Study of Encephalitis led by the UK Health Protection Agency (HPA; now UK Health Security Agency) [17]. The HPA study recruited prospectively 203 patients from 24 hospitals in England over a three-year period (2005–2008). Entry criteria were those admitted to hospital with encephalopathy (defined as altered consciousness that persisted for longer than 24 hours, including lethargy, irritability, or a change in personality and behaviour) and two or more of the following:

- Fever on admission or a history of fever (≥38.C) during the presenting illness
- Seizures and/or focal neurological findings
- Cerebrospinal fluid (CSF) pleocytosis (more than four white blood cells per μL)
- Electroencephalographic (EEG) findings indicative of encephalitis
- Abnormal neuroimaging suggestive of encephalitis

These criteria are consistent with the WHO AES definition [3].

**Fig. 1:**
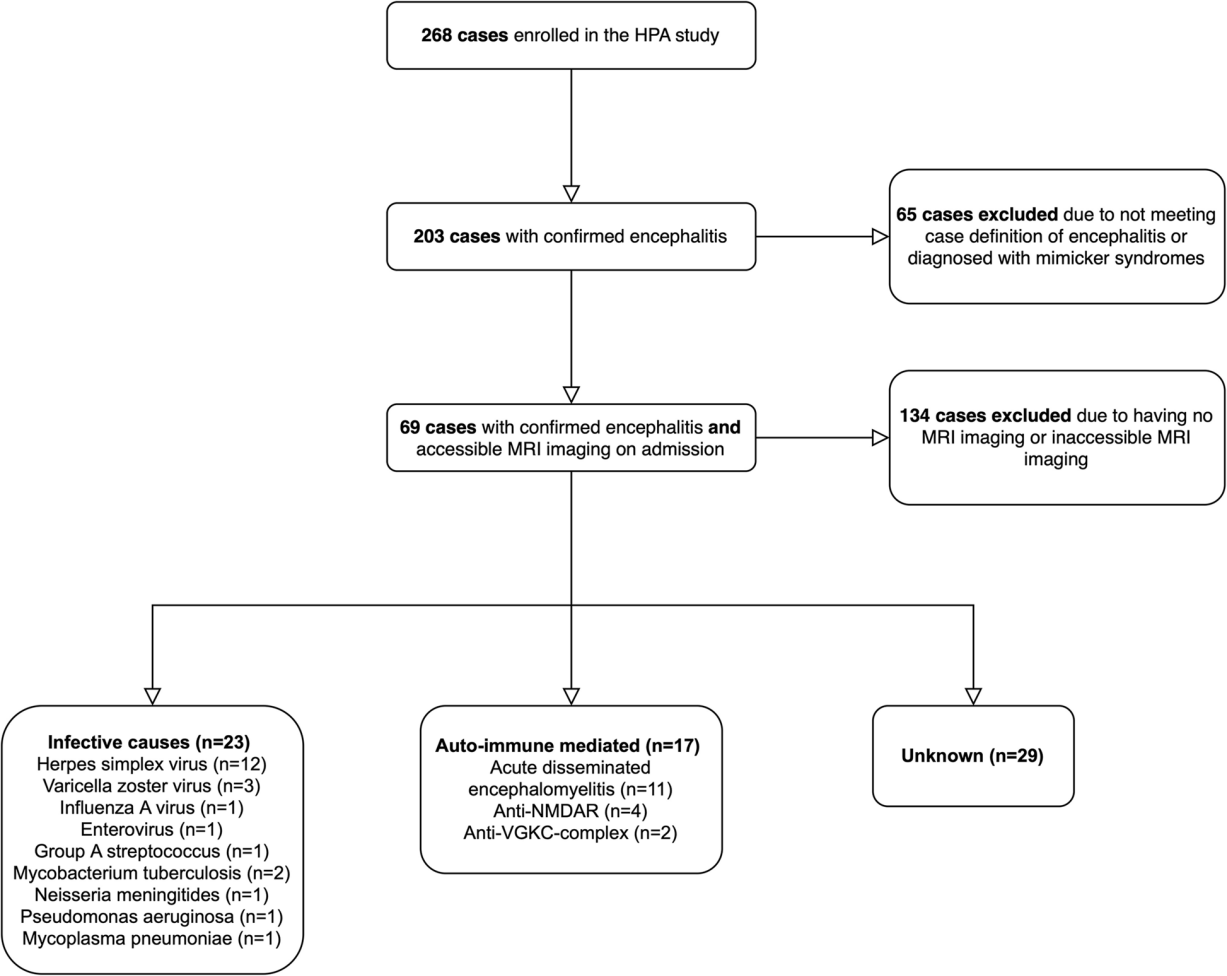
Flow diagram showing study population and aetiologies of encephalitis; HPA = Health Protection Agency’s Prospective Aetiological Study of Encephalitis; NMDAR = N-methyl-D-aspartate receptor; VGKC = Voltage-gated potassium channel; MRI = Magnetic resonance imaging

Patients were included into our retrospective study if they fulfilled the following criteria: confirmed diagnosis of encephalitis and accessible MRI scan within 3 days of admission (Fig. 1).

### Clinical information

Demographic and clinical data were collected from documentation in the HPA case files. This included age, sex, Glasgow coma scale (GCS) score on admission, duration of symptoms prior to admission, and aetiology. Aetiology was determined through analysis of the relevant clinical documentation and CSF results by the study team according to predefined definitions [17]. Radiology reports of the imaging were collected and used to corroborate findings during volumetric analysis of oedema.

The outcomes studied were:

- Seizures which occurred at any time point during inpatient stay and after MRI imaging
- Glasgow outcome score (GOS) at 6 months post-discharge (with a score of 1–3 defined as a ‘poor’ outcome)

### Regional and volumetric analysis of cerebral oedema

To be included in this study, each patient required a 2D T2-weighted and/or T2-weighted fluid attenuated inversion recovery (FLAIR) MRI of the brain. MRI scans that were acquired in axial and coronal planes were included. All scans were acquired as part of the routine clinical care of patients on admission. Cerebral oedema was defined as hyperintense T2-weighted voxels [18].

**Fig. 2:**
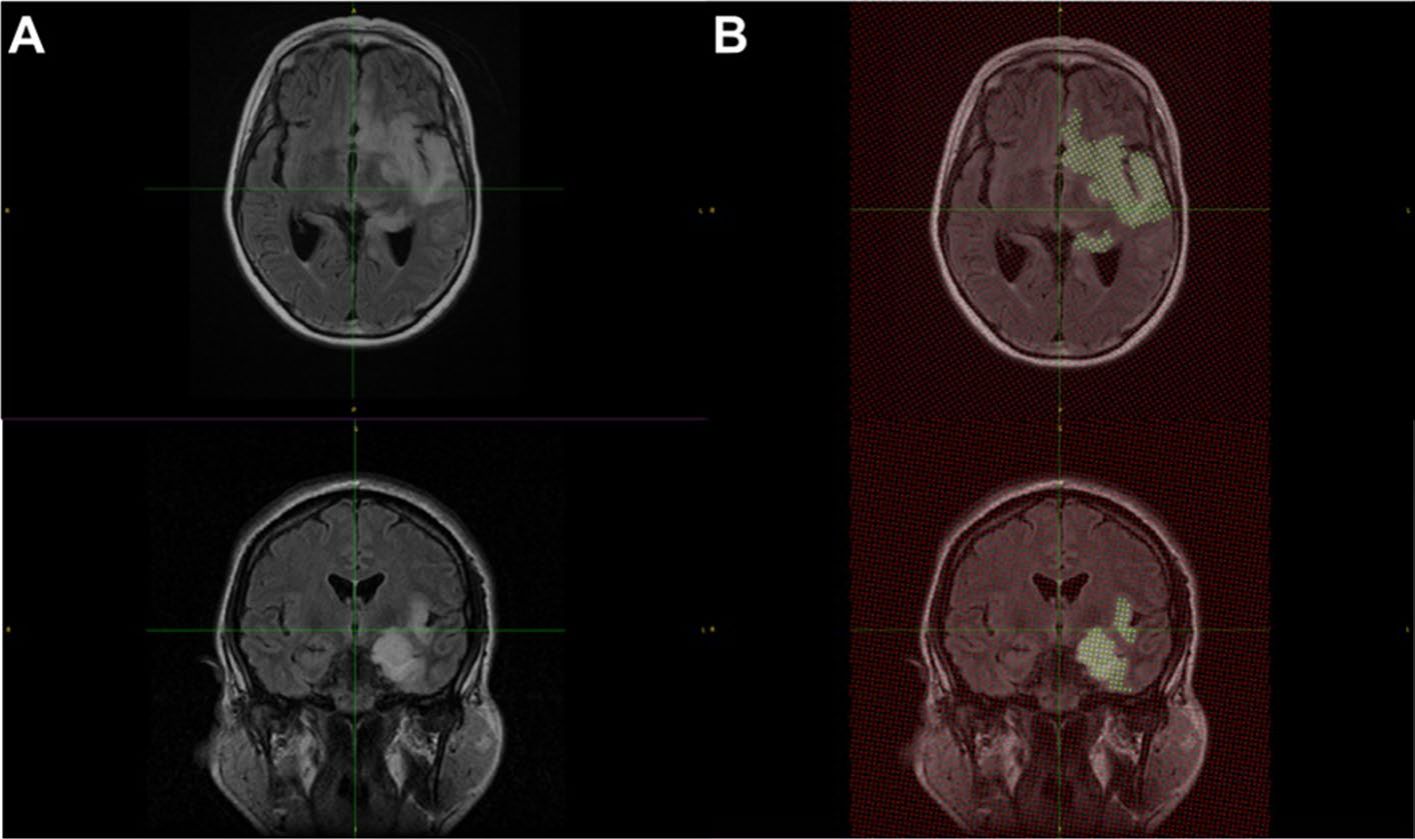
**A** MRI coronal and axial slices showing areas of cortical oedema in patients with acute encephalitis syndrome, **B** The same MRI slices analysed on EasyMeasure using point counting with stereology; yellow dots signify areas of oedema and are stereologically analysed to create a volume of oedema

Volumetric analysis was conducted by two independent blinded investigators using the Cavalieri method of stereology with point counting, an unbiased and reproducible technique for volume estimation of brain compartments on MRI with high precision [19]. Stereological analysis was performed using *EasyMeasure* software [20], as previously applied in other stereological studies of compartmental brain volumes [21]. In *EasyMeasure*, scans were displayed with a square grid of probe points superimposed in a random position. Points which intersected T2-weighted hyperintensities on MRI slices were demarcated (Fig. 2). The volume of oedema in millimetres^3^ (mm^3^) was calculated from the total number of points counted on consecutive MRI sections through the software algorithm.

The MRIs of the brain were divided into the following cortical and subcortical structures to characterise the location of oedema: frontal lobe, temporal lobe, parietal lobe, and occipital lobe, brain stem, cerebellum, basal ganglia, internal capsule, and thalamus [22]. Inter-observer reliability analyses were conducted by blinded investigators to reduce observer bias.

### Statistical analysis

Statistical analysis was conducted using SPSS (v27.0, IBM Corp., USA). When analysing volume of oedema, due to the wide range of oedema values a logarithmic transformation was conducted to account for our zero-inflated skewed non-parametric data to aid the accessibility of graphical representations (the transformation is defined in Supplement 1). Intra- and inter-observer reliability analysis of the two investigators calculating volume of oedema was conducted using a two-way mixed interclass correlation coefficient on raw data prior to log transformation. A correlation co-efficient greater than 0.90 was deemed acceptable. We reported mean and standard deviations (SD) for parametric data, and median with interquartile range (IQR) for non-parametric data. Categorical variables were compared using the Chi-square test, whilst Mann-Whitney U rank sum test was used for comparison between continuous variable groups. We assigned missing information with the multiple imputations method and chose the mean or median of these values for final calculations. Logistical regression was used to study the association between independent variables and seizures or outcome, with associations assessed by odds ratios (OR) with 95% confidence intervals (CI). The terms selected were variables which were found to be significant in univariate analysis, which were then controlled for in a multivariate backwards stepwise regression to test for independent effects. In multivariate modelling for seizure activity, we used GCS as the covariate and the presence of oedema in a structure and the volume of oedema in the same structures were assessed as collinear variables. In the multivariate model for outcomes, the co-variate used was aetiology. Multivariate results were reported as adjusted odds ratios (aOR). All tests were two sided, with the threshold of significance set at *p* < 0.05.

Due to small sample size, our study did not correct for multiple comparisons.. Nevertheless, we did endeavour to address risks associated with Type 1 errors through blinded assessment by independent imaging analysts to quantify volume of cerebral oedema and by focusing our analysis on the primary hypotheses that the volume of cortical, especially temporal, oedema would be most strongly associated with seizures and poor outcome [7, 11]. All statistical tests conducted are provided in the online supplementary materials.

## Results

### Baseline demographics and clinical characteristics

From 203 patients in the first cohort, 69 (25.7%) met the inclusion criteria with a mean (SD) age of 31.8 (23.2) years and 37 (53.6%) were female (Table 1). Median (IQR) GCS on admission was 13 (8–15) and median duration of symptoms prior to admission was 7 (4–16) days. The most common aetiology identified was auto-immune mediated encephalitis, which was present in 17/69 (24.6%) cases. This was followed by herpes simplex virus (HSV) in 12/69 (17.4%) cases and other infective causes were identified in 11/69 (15.9%) patients. Aetiological diagnosis of encephalitis was not reached in 29/69 (42.0%) patients. Overall, 41/69 (59.4%) patients had acute seizures, whilst 21/69 (30.4%) had a poor outcome at six-month follow-up.

**Table 1:** Patient characteristics of our study

	***n*** = 69
Mean age (SD)	31.8 (22.9)
Female (%)	37 (53.6%)
Median GCS on admission (IQR)	13 (8–15)
Median duration of symptoms in days prior to admission (IQR)	7 (4–16)
Visible oedema on MRI (%)	44 (63.8%)
Aetiology (%)	
Unknown	29 (42.0%)
HSV	12 (17.4%)
Other infective cause	11 (15.9%)
Auto-immune mediated	17 (24.6%)
Seizures (%)	41 (59.4%)
Glasgow outcome score six-months post-discharge (%)	
1 – Death	4 (5.8%)
2 – Persistent vegetative state	0 (0.0%)
3 – Severe disability^a^	17 (24.6%)
4 – Moderate disability^b^	13 (18.8%)
5 – Good recovery	34 (50.7%)

*SD* Standard deviation, *GCS* Glasgow coma scale, *IQR* Interquartile range, *HSV* Herpes simplex virus, *MRI* Magnetic resonance imaging

^a^ Permanent need of help with activities of daily living

^b^ No need for assistance in everyday life and employment is possible but may require special equipment for daily activities

### MRI regional and volumetric analysis

**Fig. 3:**
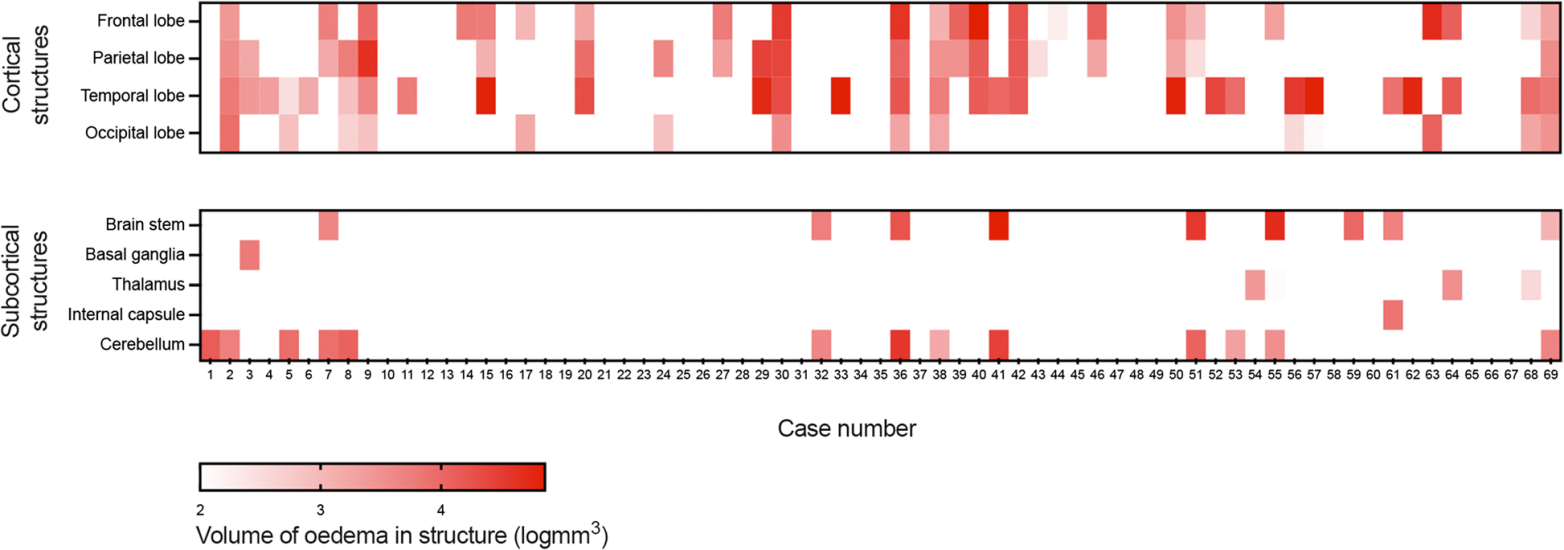
Heat map illustrating regional analysis of oedema on Magnetic resonance imaging (MRI) in our cohort overall

Regional analysis of MRI showed cerebral oedema in 44/69 (63.8%) patients, predominantly affecting cortical structures in 40/44 (90.9%). Of these, 15/40 (37.5%) patients had temporal lobe oedema only, whilst 10/40 (25.0%) had frontal lobe oedema only and 13/40 (32.5%) had both frontal and temporal oedema. Only 2/40 (5.0%) had partial lobe oedema in isolation. No patients had isolated occipital lobe oedema. Oedema in the subcortical structures was found in 19/44 (43.2%) cases (Fig. 3). The mean (SD) number of structures with visible oedema on MRI was 2.51 (2.47).

Pre-analysis volumetric reliability assessment of raw data identified a high degree of consistency between blinded investigators with an average (95% CI) interclass correlation of 0.96 (0.83–0.99, *p* < 0.01) within investigators and 0.97 (0.81–0.99, *p* < 0.01) between investigators.

The median (IQR) volume of oedema in the brain was 3865 (0–23,152) mm^3^. The median volume of oedema in the cortical structures was 1902 (0–17,449) mm^3^, whilst subcortical structures had a median volume of 0 (0–761) mm^3^ (Fig. 3).

### MRI analyses and acute seizure activity in patients with acute encephalitis syndrome

Oedema in the cortical structures was seen in a greater proportion of patients who had seizures compared to those who did not (30/41 [73.2%] vs 10/28 [35.7%], Chi-squared *p* < 0.01). Just over half of those who had seizures also had observable temporal lobe oedema, which was significantly higher than the no seizure group (21/41 [51.2%] vs 7/28 [25.0%], *p* = 0.03). The mean (SD) number of structures with observable oedema in those who had seizures was 3.12 (2.41), whilst 1.61 (2.02) structures had oedema in those who did not have seizures (Chi-squared *p* = 0.01).

**Fig. 4:**
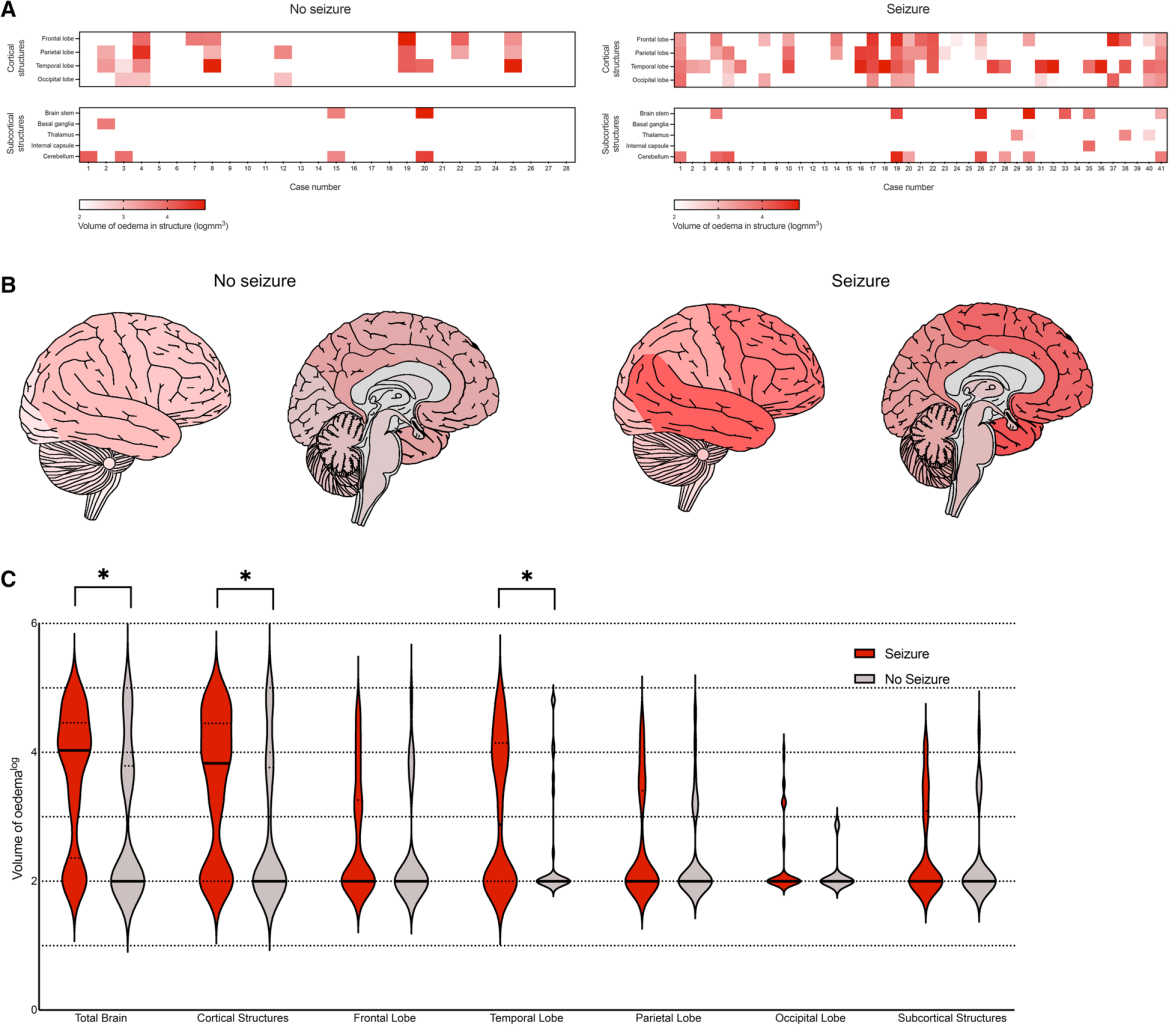
**A** Heat map illustrating regional analysis of oedema on magnetic resonance imaging (MRI) in seizure and no seizure groups; **B** regional analysis of oedema in seizure and no seizure groups shown schematically with darker red illustrating regions where higher volume of oedema was identified; **C** violin plot illustrating comparison of log volume of oedema measured in brain structures in patients with (red) and without seizures (grey); black lines illustrate median, whilst dotted lines illustrate 25th and 75th percentiles; Mann-Whitney U test was used to assess significance; * = *p* < 0.05

The overall median [IQR] volume of cerebral oedema was significantly increased in those who had a seizure as opposed to those who did not (10,527 [138–28,518] mm^3^ vs 0 [0–6067] mm^3^, Mann-Whitney U *p* = 0.02) (Fig. 4). In addition, the volume of oedema in cortical structures was significantly higher in those with seizures (6625 [0–27,884] mm^3^ vs 0 [0–5778] mm^3^, *p* = 0.01). Greater volumes of oedema of the temporal lobe were identified in those with seizures than those without (653 [0–13,915] mm^3^ vs 0 [0–131] mm^3^, *p* = 0.03).

### MRI analyses and neurological outcomes in patients with acute encephalitis syndrome

**Fig. 5:**
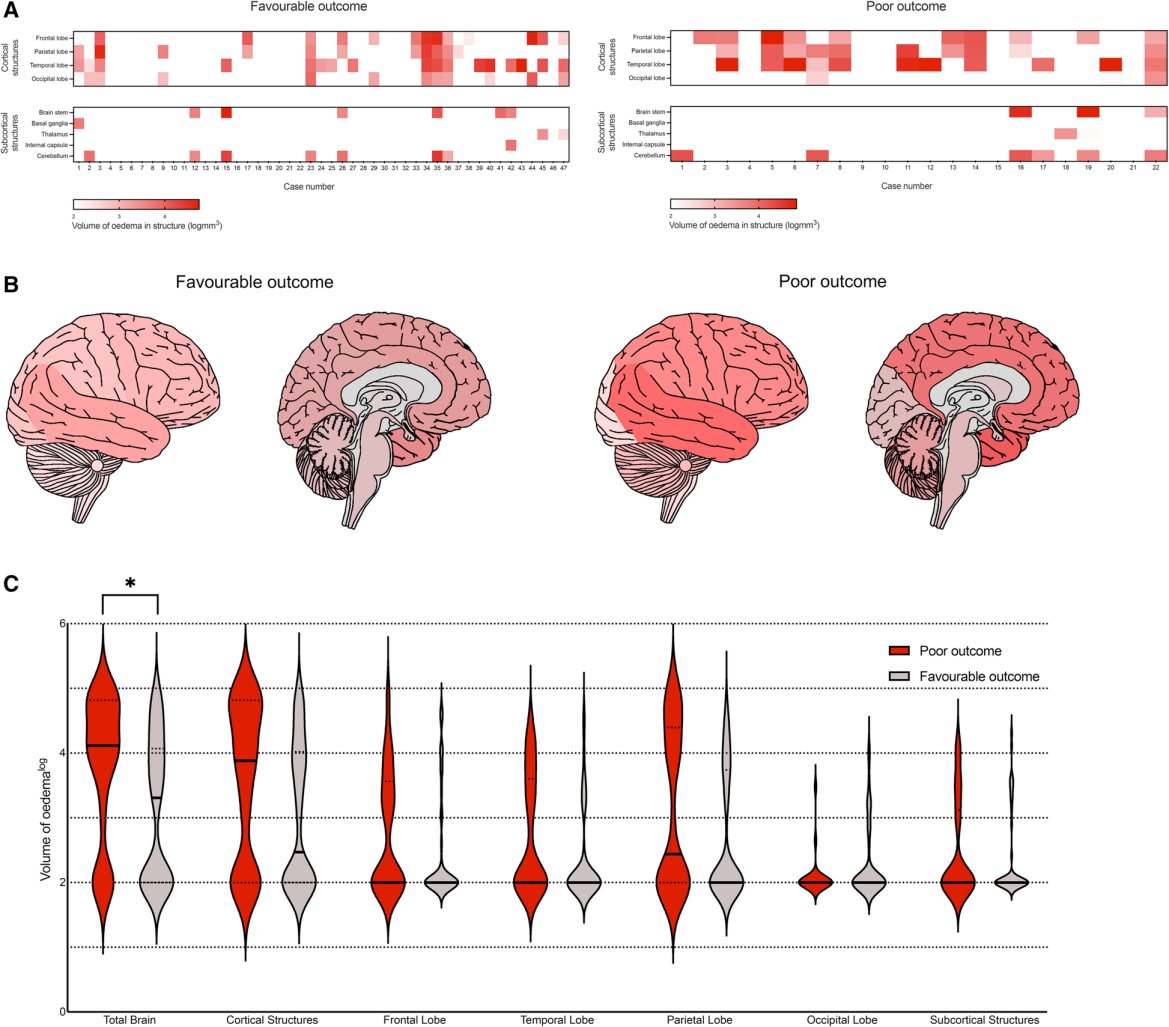
**A** Heat map illustrating regional analysis of oedema on Magnetic resonance imaging (MRI) in poor and favourable outcome groups; **B** regional analysis of oedema in poor and favourable groups shown schematically with darker red illustrating regions where higher volume of oedema was identified; **C** violin plot illustrating comparison of log volume of oedema measured in brain structures in patients with poor (red) and favourable (grey) outcomes; black lines illustrate median, whilst dotted lines illustrate 25th and 75th percentiles; Mann-Whitney U test was used to assess significance; * = *p* < 0.05

Greater median (IQR) total volume of oedema was seen in patients with poor outcomes when compared to those with favourable outcomes (12,884 [1720-67,777] mm^3^ vs 2157 [0–11,889] mm^3^; Mann-Whitney U *p* = 0.02) (Fig. 5). We found no other statistically significant results when comparing volumes or regions of oedema and the outcome of the patient (all statistical tests are shown in Supplement 2).

### Risk factors for seizure activity

On univariate analysis, higher GCS on admission was a strong negative predictor for seizure activity (OR [95% CI] 0.67 [0.52–0.85], *p* < 0.01) (Table 2). Of neuroimaging variables, the presence of oedema in the cortical structures was the strongest predictor of seizure activity in univariate analysis (OR 4.91 [1.74–14.39], *p* < 0.01). All other variables were not significant in univariate analysis. When conducting multivariable regression, higher GCS remained a negative predictor of seizures (aOR 0.61 [0.46–0.83], *p* < 0.01). Adjusting for GCS on admission as an interaction, the presence of cortical oedema was a risk factor for seizure activity (aOR 5.48 [1.62–18.51], *p* = 0.01) and increased volume of oedema in the cortical structures increased the risk of seizures (aOR 1.90 [1.12–3.21], *p* = 0.02) specifically.

**Table 2:** Univariate regression modelling of variables with seizure activity and poor outcomes; volumes of oedema were log transformed prior to regression modelling

	Univariate analysis		Multivariate analysis	
	Odds ratio for seizure activity (95% CI)	***P*** value	Odds ratio for seizure activity adjusting for admission GCS (95% CI)	***p*** value
**Clinical variables**				
Age	0.99 (0.97–1.01)	0.390	–	**–**
Sex (male)	1.28 (0.49–3.35)	0.618	–	**–**
GCS	0.67 (0.53–0.84)	< 0.001**	0.61 (0.46–0.83)	0.001**
Duration of symptoms in days	1.01 (0.99–1.02)	0.424	–	**–**
**Aetiology**				
Unknown	1.56 (0.58–4.17)	0.381	–	**–**
Herpes simplex virus	2.34 (0.57–9.58)	0.236	–	**–**
Other infective cause	0.79 (0.22–2.89)	0.726	–	**–**
Auto-immune mediated	0.37 (0.12–1.14)	0.083	–	**–**
**Regional analysis**				
Visible oedema on MRI	4.74 (1.65–13.58)	0.004**	0.63 (0.05–8.31)	0.723
Presence of cortical oedema	4.91 (1.79–14.39)	0.003**	5.48 (1.62–18.51)	0.006*
Presence of frontal lobe oedema	2.60 (0.90–8.29)	0.088	–	**–**
Presence of temporal lobe oedema	3.15 (1.14–9.50)	0.033*	0.67 (0.09–4.46)	0.674
Presence of parietal lobe oedema	1.56 (0.54–4.75)	0.419	–	**–**
Presence of occipital lobe oedema	3.06 (0.84–14.61)	0.113	–	**–**
Presence of subcortical oedema	2.39 (0.78–8.30)	0.142	–	**–**
Presence of cerebellum oedema	1.69 (0.49–6.83)	0.427	–	**–**
Number of structures with oedema	1.36 (1.09–1.76)	0.012*	1.10 (0.72–1.68)	0.646
**Volumetric analysis**				
Total volume of oedema	1.75 (1.13–2.81)	0.015*	0.72 (0.16–3.19)	0.665
Cortical volume of oedema	1.79 (1.15–2.89)	0.013*	1.90 (1.12–3.21)	0.017*
Volume of frontal lobe oedema	1.36 (0.76–2.43)	0.300	–	**–**
Volume of temporal lobe oedema	1.77 (1.06–2.95)	0.029*	1.02 (0.38–2.72)	0.966
Volume of parietal lobe oedema	1.27 (0.68–2.37)	0.459	–	**–**
Volume of occipital lobe oedema	3.08 (0.84–11.31)	0.089	–	**–**
Volume of subcortical oedema	1.49 (0.72–3.12)	0.286	–	**–**
Volume of cerebellum oedema	1.28 (0.52–3.12)	0.589	–	**–**

*CI* Confidence intervals

* = *p* < 0.05

** = *p* < 0.005

### Risk factors for poor outcome

On univariate analysis, a diagnosis of HSV encephalitis was identified as a risk factor for poor outcome at six-month follow up (OR 3.92 [1.08–14.20], *p* = 0.038) (Table 3). Worse outcomes were seen in patients with higher volumes of overall cerebral oedema (OR 1.73 [1.08–2.92], *p* = 0.03). All other variables were not significant in univariate analysis. When conducting multivariate analysis and adjusting for aetiology as an interaction, an increased volume of oedema in the brain was a remained associated with an adverse neurological outcome (aOR 1.73 [1.06–2.83], *p* = 0.03). However, HSV as an aetiology was no longer a predictor of poor outcome (aOR 2.28 [0.53–9.84], *p* = 0.27). This implies that it is the presence of oedema, as opposed to a particular aetiology, which was the strongest predictor of poor outcome in this cohort.

**Table 3:** Univariate and multivariate regression modelling of variables with poor outcomes; volumes of oedema were log transformed prior to regression modelling

	Univariate analysis		Multivariate analysis	
	Odds ratio for poor outcome (95% CI)	***P*** value	Adjusted odds ratio for poor outcome adjusting for aetiology (95% CI)	***p*** value
**Clinical variables**				
Age	1.01 (0.99–1.04)	0.215	–	–
Sex (male)	1.38 (0.50–3.86)	0.538	–	–
GCS	0.87 (0.74–1.03)	0.114	–	–
Duration of symptoms in days	1.00 (0.99–1.02)	0.587	–	–
Seizure as inpatient	2.35 (0.78–7.05)	0.128	–	–
**Aetiology**				
Unknown	0.71 (0.25–2.01)	0.515	–	–
Herpes simplex virus	3.92 (1.08–14.20)	0.038*	2.28 (0.53–9.84)	0.271
Other infective cause	0.42 (0.08–2.14)	0.298	–	–
Auto-immune mediated	0.86 (0.26–2.83)	0.801	–	–
**Regional analysis**				
Visible oedema on MRI	2.52 (0.84–8.70)	0.116	–	–
Presence of cortical oedema	1.89 (0.67–5.73)	0.243	–	–
Presence of frontal lobe oedema	2.18 (0.76–6.34)	0.148	–	–
Presence of temporal lobe oedema	1.77 (0.63–4.99)	0.278	–	–
Presence of parietal lobe oedema	2.73 (0.93–8.15)	0.068	–	–
Presence of occipital lobe oedema	0.52 (0.11–1.90)	0.353	–	–
Presence of subcortical oedema	1.361 (0.43–4.10)	0.587	–	–
Presence of brain stem oedema	1.08 (0.21–4.57)	0.920	–	–
Presence of cerebellum oedema	2.14 (0.61–7.46)	0.226	–	–
Number of structures with oedema	1.15 (0.93–1.44)	0.198	–	–
**Volumetric analysis**				
Total volume of oedema	1.73 (1.08–2.92)	0.027*	1.73 (1.06–2.83)	0.027*
Cortical volume of oedema	1.53 (0.98–2.46)	0.068	–	–
Volume of frontal lobe oedema	1.46 (0.83–2.58)	0.185	–	–
Volume of temporal lobe oedema	1.51 (0.94–2.45)	0.087	–	–
Volume of parietal lobe oedema	1.75 (0.94–3.31)	0.077	–	–
Volume of occipital lobe oedema	0.39 (0.07–1.25)	0.176	–	–
Volume of subcortical oedema	1.26 (0.61–2.55)	0.518	–	–
Volume of brain stem oedema	1.07 (0.38–2.65)	0.880	–	–
Volume of cerebellum oedema	1.58 (0.65–3.81)	0.298	–	–

*CI* Confidence intervals

* = *p* < 0.05

## Discussion

Acute seizures are a significant cause of morbidity in AES, and most survivors suffer from long-term sequelae [11, 12, 23]. Identifying factors associated with seizures in these patients can help to recognise those at highest risk to direct acute management, such as ensuring provision of care in a setting capable of managing seizures (and subsequent status epilepticus) and close monitoring (potentially through EEG). Early recognition of patients likely to have adverse outcomes can enable prompt referral to neuro-rehabilitation services. By utilising stereological analysis, this study identified that higher volumes of oedema, especially when involving cortical structures and the temporal lobe, are associated with both an increased risk of acute seizure activity and adverse long-term outcomes in patients with AES.

Inpatient seizures were common in our cohort and observed in 59% of patients, which correlates with the estimated incidence of acute seizures in encephalitis [11]. In our multivariate analysis, the presence of oedema in the cortical structures specifically increased the risk of acute seizures. This is consistent with previous studies that found cortical structure involvement on MRI to be a significant risk factor for the development of seizures in encephalitis [7, 14, 24]. The association with cortical structures oedema and seizure risk has been further observed in other intracranial pathologies, including malignancy, ischaemia events and other CNS infections [25–28]. Several pro-inflammatory cytokines likely play a key role in lowering the seizure threshold in AES through excitatory neurotransmitter release and the generation of cytotoxic oedema and vasogenic oedema [29–31]. This is particularly seen when inflammation involves known epileptogenic areas of brain parenchyma (such as the cortical structures) [8]. More extensive inflammation in AES reflected in greater volumes of observable cerebral oedema may further lower this seizure threshold, and we found that greater volumes of oedema in the cortical structures increased the risk of seizure activity. Visualisation of oedema on MRI could therefore provide an in-vivo assessment of the pathological excitatory activity, allowing it to act as a useful indicator for seizure risk.

Other predisposing factors for seizures in AES include reduced GCS, abnormal EEG and specific aetiologies (such as Leucine-rich glioma-inactivated 1 and HSV encephalitis) [32–35]. In our study, impaired GCS on admission was a pronounced predictor of seizure activity. Our multivariate findings suggest presence of oedema on MRI can act as an additional independent indicator for identification of patients at high-risk of acute seizures. Neuroimaging should be performed as soon as possible after an LP in cases of suspected AES, with MRI being the preferred modality [23, 36]. By recognising the presence of oedema through visualising T2 hyperintensities on MRI, non-specialist clinicians can identify patients who may be at risk of seizure activity. Identification of at-risk patients can direct early interventions such as intravenous cannula, intensive care unit notification and observation, and EEG monitoring. There is a paucity of trials assessing routine anti-seizure medication as prophylaxis in AES [23, 37]. Identifying those at high risk would allow for stratification of patients in studies evaluating the efficacy of initiating prophylactic antiseizure medications in AES. Our study assessed acute inpatient seizures only and therefore cannot describe the relationship between cerebral oedema in the acute phase and subsequent development of post-encephalitis epilepsy. However, there is evidence that early seizures are strongly associated with further seizures and long-term epilepsy [38].

A proportion of our cohort had no confirmed underlying aetiology. This reflects what is seen in the clinically in the acute phase of hospital admission, where aetiological diagnosis is still unknown. However, it limits the extent of subgroup-analysis. The underlying aetiology of encephalitis is known be a determinant of both seizure frequency and patterns of MRI involvement [11, 39]. For example, HSV and acute disseminated encephalomyelitis (ADEM) frequently have extensive MRI involvement. However, seizures are less frequently observed in ADEM (which is predominantly a white-matter disease) compared to HSV encephalitis (which results in both grey and white matter involvement) [17]. Oedema volume may better predict risk of seizures in some aetiologies of encephalitis than others.

Furthermore, many patients in our cohort were admitted with impaired consciousness, which may signify peri-ictal periods on admission. Peri-ictal MRI abnormalities that develop immediately after seizures have been well described and these neuroimaging changes in the postictal period may represent the effect of the seizure activity, rather than its structural cause [40, 41]. It is theorised that seizures beget seizures [42], and the potential utility of cerebral oedema as a predictor for seizure activity may rest in its role as a marker for prior seizure activity [43]. To address this, we adjusted the volumetric data by GCS on admission to mitigate effects in any potentially post-ictal patients. Despite this adjustment presence and volume of cerebral oedema were both significantly associated with acute seizure activity.

Previous studies have found the presence of abnormal MRI findings to be associated with poor functional outcome, however, the role of oedema in this association has not been determined [44–47]. Our study identified that larger volumes of vasogenic oedema in the brain increased risk of poor neurological outcome at six-months – and remained a risk factor when controlling for HSV as an aetiology, suggesting that oedema may play a key role in the poor outcomes seen in AES overall. The pro-inflammatory state in AES promotes cytokine networks, including interferon γ and interleukin 1 and 6, which are associated with a worse prognosis [48, 49]. This initial cytotoxic injury can propagate blood brain barrier breakdown and formation of vasogenic oedema [50]. Increased cerebral oedema may therefore signify significant brain inflammation and neuronal damage, suggesting its potential as prognostic marker. Patients with larger volumes of oedema on MRI may warrant closer observation on discharge for secondary neurological sequelae.

### Strengths and limitations

The main strength of our study was the inclusion of a substantial cohort of patients with AES of multiple aetiologies. We utilised a robust method of volumetric analysis which allows for determination of specific amount of oedema in brain structures. This method was tested for reliability, and we obtained a high degree of correlation between blinded investigators, further demonstrating the feasibility of stereological MRI evaluation. We acknowledge that log transformation of data may potentially increase the degree of interobserver agreement. However, in this study log transformed data were not used to assess interobserver variability and similarly high rates of interobserver agreement have been reported in volumetric studies of oedema in HSV encephalitis [16].

We acknowledge that several conditions could result in a bias in reporting of seizures in those admitted with AES. As we used clinical record forms to determine the occurrence of a seizure, subclinical presentations may have not been reported. Specific seizure types such as focal seizures or non-convulsive status may be under-reported. Furthermore, those with low GCS may be more likely to undergo prolonged EEG monitoring, and this may increase the chance of detecting seizures. Treatment effects may also affect reporting of seizures. When analysing cerebral oedema, we only studied gross visible oedema on T2 or FLAIR imaging. Other types of oedema such as cytotoxic oedema would not be quantifiable in these imaging sequences. The MRIs we studied occurred on admission and hence the volume of observed oedema may be underestimated if performed early or prior to ongoing disease progression. Future studies should focus on the impact of the frequency of seizures prior to and during the inpatient admission and the development of symptomatic epilepsy following AES in the long term relative to the evolution of cerebral oedema over time. In light of the International League Against Epilepsy recommendations, patients should be followed up for at least 1 year to enable evaluation for chronic post-AES epilepsy [51]. Finally, corrections for multiple comparisons were not attempted in our study due to sample size; future larger studies would aid in corroborating our findings from the univariate analysis.

## Conclusion

In this analysis of a multi-centre cohort of patients presenting with AES, larger volumes of oedema on admission MRI, particularly in cortical structures, was independently associated with risk of acute inpatient seizures. Increasing volumes of overall cerebral oedema was also associated with poorer outcomes at six-month follow-up. Early identification of patients at greatest risk of acute seizure activity and subsequent morbidity through MRI findings may help clinicians to improve the acute and long-term care of patients with AES.

## Supplementary Information

**Additional file 1: Supplement 1.** Log transformation of volumetric data. **Supplement 2.** statistical tests on volumetric data.

## Data Availability

The data that support the findings of this study are available from UK Health Protection Agency’s (now UK Health Security Agency) Aetiological Study of Encephalitis but restrictions apply to the availability of these data, which were used under license for the current study, and so are not publicly available. The de-identified data that support the findings of this study are available are available from the corresponding author on reasonable request but researchers should first provide a methodologically sound proposal for approval by the UK Health Security Agency, Virus Reference Department prior to requests.
