# Correction: Increased volume of cerebral oedema is associated with risk of acute seizure activity and adverse neurological outcomes in encephalitis – regional and volumetric analysis in a multi-centre cohort

**DOI:** 10.1186/s12883-022-02995-6

**Published:** 2022-12-05

**Authors:** Ali M. Alam, Jian P. K. Chen, Greta K. Wood, Bethany Facer, Maneesh Bhojak, Kumar Das, Sylviane Defres, Anthony Marson, Julia Granerod, David Brown, Rhys H. Thomas, Simon S. Keller, Tom Solomon, Benedict D. Michael

**Affiliations:** 1 Department of Clinical Infection Microbiology and Immunology, Institute of Infection, Veterinary, and Ecological Science, University of Liverpool, Liverpool, UK; 2 The NIHR Health Protection Research Unit for Emerging and Zoonotic Infection, Liverpool, UK; 3 Barts Health NHS Trust, London, UK; 4 Department of Pharmacology and Therapeutics, Institute of Systems, Molecular and Integrative Biology, University of Liverpool, Liverpool, UK; 5 Department of Neuroradiology, The Walton Centre NHS Foundation Trust, Liverpool, UK; 6 Tropical and Infectious Diseases Unit, Liverpool University Hospitals NHS Trust, Liverpool, UK; 7 Department of Neurology, The Walton Centre NHS Foundation Trust, Liverpool, UK; 8 Independent Scientific Consultant, formerly of Public Health England, London, UK; 9 UK Heath Security Agency, 61 Colindale Avenue, London, UK; 10 Translational and Clinical Research Institute, Faculty of Medical Sciences, Newcastle University, Newcastle upon Tyne, UK

**Correction: BMC Neurol 22, 412 (2022)**

**https://doi.org/10.1186/s12883-022-02926-5**

Following publication of the original article [1], the authors would like to expand the given names for all authors as the current presentation are all in initials. The following are expanded given names:

Ali M. Alam

Jian P. K. Chen

Greta K. Wood

Bethany Facer

Maneesh Bhojak

Kumar Das

Sylviane Defres

Anthony Marson

Julia Granerod

David Brown

Rhys H. Thomas

Simon S. Keller

Tom Solomon

Benedict D. Michael

The original article [1] has been updated.
