# Supplementary material for: Increased volume of cerebral oedema is associated with risk of acute seizure activity and adverse neurological outcomes in encephalitis – regional and volumetric analysis in a multi-centre cohort
Source: BMC Neurol. 2022 Nov 7;22:412. doi: 10.1186/s12883-022-02926-5 (PMC9639313; doi:10.1186/s12883-022-02926-5)
Supplement: Supplementary file 1 — Additional file 1: Supplement 1. Log transformation of volumetric data. Supplement 2. statistical tests on volumetric data. [file 12883_2022_2926_MOESM1_ESM.docx]

**Supplement 1 – Log transformation of volumetric data**

$$\boldsymbol{Volume of oedema=}\boldsymbol{Log}\boldsymbol{10}\left( \boldsymbol{Volume in m}\boldsymbol{m}^{\boldsymbol{3}}\boldsymbol{+100 m}\boldsymbol{m}^{\boldsymbol{3}} \right)$$

$$\boldsymbol{e.g.}$$

$$\boldsymbol{volume calculated=1558.29}\boldsymbol{m}\boldsymbol{m}^{\boldsymbol{3}}$$

$$\boldsymbol{\to Log}\boldsymbol{10}\left( \boldsymbol{1558.29}\boldsymbol{m}\boldsymbol{m}^{\boldsymbol{3}}\boldsymbol{+100}\boldsymbol{m}\boldsymbol{m}^{\boldsymbol{3}} \right)$$

$$\boldsymbol{\to3.21965786}$$

$$\boldsymbol{no oedema=0}\boldsymbol{m}\boldsymbol{m}^{\boldsymbol{3}}$$

$$\boldsymbol{\to Log}\boldsymbol{10}\left( \boldsymbol{0}\boldsymbol{m}\boldsymbol{m}^{\boldsymbol{3}}\boldsymbol{+100}\boldsymbol{m}\boldsymbol{m}^{\boldsymbol{3}} \right)$$

$$\boldsymbol{\to2.00000000}$$

**Supplement 2 – statistical tests on volumetric data**

| **Region** | **Median volume of oedema in mm^3^ in seizure group (IQR)** | **Median volume of oedema in mm^3^ in no seizure group (IQR)** | **p value (Mann-Whitney U test)** |
| --- | --- | --- | --- |
| Total brain | 10646 (168-28202) | 0 (0-6067) | **0.015** |
| Cortical structures | 7026 (0-27252) | 0 (0-5778) | **0.010** |
| Frontal lobe | 0 (0-1647) | 0 (0-0) | 0.162 |
| Parietal lobe | 0 (0-2237) | 0 (0-818) | 0.388 |
| Temporal lobe | 327 (0-14854) | 0 (0-130) | **0.030** |
| Occipital lobe | 0 (0-280) | 0 (0-0) | 0.082 |
| Subcortical structures | 0 (0-1480) | 0 (0-0) | 0.190 |
| Cerebellum | 0 (0-175) | 0 (0-0) | 0.508 |

| **Region** | **Median volume of oedema in mm^3^ in poor outcome group (IQR)** | **Median volume of oedema in mm^3^ in favourable outcome group (IQR)** | **p value (Mann-Whitney U test)** |
| --- | --- | --- | --- |
| Total brain | 12884 (1720-67777) | 2157 (0-11889) | **0.016** |
| Cortical structures | 7567 (0-67777) | 138 (0-10498) | 0.054 |
| Frontal lobe | 0 (0-2751) | 0 (0-457) | 0.234 |
| Parietal lobe | 0 (0-3379) | 0 (0-49) | 0.088 |
| Temporal lobe | 327 (0-441756) | 0 (0-5427) | 0.087 |
| Occipital lobe | 0 (0-65) | 0 (0-0) | 0.344 |
| Subcortical structures | 0 (0-1886) | 0 (0-297) | 0.442 |
| Cerebellum | 0 (0-1131) | 0 (0-0) | 0.185 |
